# Some Reminiscences and a Forecast
*An address delivered to the Bristol Medico-Chirurgical Society on Wednesday, 11th February, 1931.


**Published:** 1931

**Authors:** Ewen J. Maclean

**Affiliations:** Professor of Obstetrics and Gynæcology, Welsh National School of Medicine; Vice-President, British Medical Association, etc.


### The Bristol Medico-Chirurgical Journal

*" Scire est nescire, nisi id me*
*Scire alius sciret."*

SUMMER, 1931.

### SOME REMINISCENCES AND A FORECAST.*

BY

Sir Ewen J. Maclean, M.D., D.Sc. (Hon.), F.R.C.P., F.A.C.S. (Hon.), F.R.S.E.,

*Professor of Obstetrics and Gynæcology, Welsh National School of Medicine;*

*Vice-President, British Medical Association, etc.*

Bristol has always seemed to me to typify in her professional classes, her business men, and in her social endeavour that combine of solid worth, steady, unfaltering grip, whether in good times or bad, and unheralded point to point attainment, which is summed up in that great word England. We may not wholly agree with Thomas Carlyle that " The English are a dumb people. They can do great acts but not describe them " ; but there is something wholly admirable and, indeed, comforting to the more vocal and demonstrative Celtic fringe in the wise, almost parental tolerance of the predominant partner.

* An address delivered to the Bristol Medico-Chirurgical Society on Wednesday, 11th February, 1931.

F

My personal knowledge of Bristol began in the last century—I do not mean a century ago, but in 1890—when I was advised to gain some knowledge of general practice by accepting an assistantship in the Lawrence Hill district, and I recall that my then very youthful appearance created some difficulty in my being received as a substitute for my chief by the actively parturient ladies of the East End. In relation to this experience, not altogether rare amongst young practitioners, a senior chief of mine later said to me : "Your physical make-up, Maclean, is like mine. For years I felt handicapped by the impression that I looked too young until one day I overheard someone say that I was too old. Believe me, young man, subjectively, there is no intermediate stage !" There was, however, some consolatory glamour in the fact that not infrequently the great W. G. Grace and I met to minister to the sick in the same street—though not in the same house, and not, of course, for the same firm.

Then I was House Surgeon for over two years at the Bristol Royal Hospital for Sick Children and Women on St. Michael's Hill, and was a member of this Society, and I trust the records show that I duly paid my subscription. Much to my profit, I was and still am a subscribing member to the *Journal*, which owes so much in its development to the late L. M. Griffiths, the memory of whose qualities and zeal are, I know, still held high among you. The Society assembled in the Old Library, the premises of which were nothing like so palatial or impressive as the beautiful building of to-day; but I would venture to claim that the functioning of the Society, in all essentials, could not be bettered. It was there that I, with other recently qualified men, met and was privileged to know the leading members of the profession, and I shall always gratefully remember the kindness and encouragement extended to us as juniors. If I may say so, as succeeding years have conferred the inevitable senior status upon me I have endeavoured to follow their example—surely one of the prime objects of our societies. To mention only a few names, I especially recall Edward Long Fox, Shingleton Smith, Markham Skerritt, Greig Smith, and last but not least, and still happily with us, Richardson Cross.

I came to Bristol from my Alma Mater, Edinburgh University, where in matters surgical antisepsis was dominant, and had not, as yet, given due place to asepsis. The smell and hiss of the carbolic spray assailed ears and nostrils in all the hospitals of the land, and those in Bristol afforded no exception. Buried sutures, including those of rather crudely prepared catgut, were coming more into vogue, and sometimes over-reaching the absorptive capacity of the tissues. Chicken-bone drainage tubes in osteotomy cases failed to disappear in the time and manner it was intended they should, and contributed to some unsatisfactory results. At the Children's Hospital I made myself responsible for the collection and preparation of the chicken bones of appropriate size and shape, and the response of my social friends became so cordial and active in the matter of collection as to create some embarrassment and even to affect appreciably the corresponding poultry market—as was jocularly suggested.

It is interesting to recall the then phase of the treatment of tuberculosis by Koch's tuberculin, which, discarding the precautions and reservations which accompanied the pronouncement by that great man, spread like putting a match to the heather, with, in many instances, devastating results. I obtained leave to witness the methods then employed in London. A very distinguished friend of mine was amongst the victims who succumbed to the unguarded enthusiasm of the movement before a more matured experience determined the limits of safety and utility.

I made many friends amongst the resident staffs of the Bristol Royal Infirmary and the Bristol General Hospital, and it was not difficult to discern the friendly rivalry between the two institutions, which was rooted in the remoter days when the feuds between Dolphinites and Anchorites were affairs of even more than wind and limb. No member of this Society, I imagine, is unacquainted with Augustin Prichard's brochure on "The Early History of the Bristol Medical School," in which it is related that the Bristol Royal Infirmary being the first of the two hospitals to obtain royal recognition, a certain Mr. Sanders, whose sympathies with one of them were not disguised, gave expression to the historic adage that "The patients who want a Sovereign remedy will now go to the Royal Infirmary; but those who want a Radical cure will go to the Hospital!" To what extent, if any, that injunction was at that time acted upon or has since been observed, it is not for a privileged guest to inquire or even to surmise.

It would appear that in those days the partisanship of the Bristol layfolk in matters medical was to some extent political, and Augustin Prichard submits—to quote again from his pamphlet — an interesting observation on their attitude toward movements other than commercial. He says: "There seem to be some slight signs abroad that the people of Bristol are beginning to shake off some of that lethargy in the matter of science, literature, and the arts which has been so conspicuous for the last half century, and to feel some care for things beside trade, commerce, and politics; and in producing this most desirable change the University College and Medical School must be allowed to have had a considerable share, and will, I hope, receive a corresponding reward in their future progress." As we view to-day the munificent benefactions of your merchant princes, and in particular those of the Wills family, we may truly claim that Mr. Prichard's faith has been lost in sight and his hope in abundant fruition.

Evolution, whether social or scientific, will not be denied its advances, and whilst for long periods it may be unseen to all but a few discerning souls, it has a way of developing a phase of full and dominant expression. In no sphere of thought and work has this been more exemplified than, during the past half century, in the art and science of medicine.

The measures of medicine have been filled to overflowing by the contributions of our own workers and those of the sister arts and sciences. New facts and new settings of truths long known have been submitted for transmuting into agencies for the prevention, alleviation and cure of disease. The mass of knowledge has grown so great that the curriculum for our students, though much extended in time, still remains congested. In practice this has become expressed in the divisions and sub-divisions of specialism, so that it is sometimes said that the specialist gets to know more and more about less and less and the general practitioner less and less about more and more. Accumulated knowledge, no doubt, supplies the *vis a tergo* in the creation of new departments of practice, but there is also a powerful *vis a fronte* in the public demand for specialist treatment. In the existence of a rising market for such services one of the dangers is the ready-made or even the reach-me-down specialist — the outcome of something like mass production out of a hasty curriculum in which a student may as early as his second year declare his suit as a prospective specialist and thereupon cause his subsequent training—largely theoretical—to be correspondingly focused, much to the detriment of that fundamental principle that all sound and justifiable specialism must be based on a thorough general training.

The General Medical Council has done much to prevent any marked degree of such untoward developments in this country, but I have personally observed them in important medical centres in other lands, where they are so rife as to cause grave concern to some of their directors. We have, in our own country, a more rational reaction to the call for the harnessing of new agencies for the diagnosis and treatment of disease, in the shape of team work in general practice, in consulting practice and in institutional service. In its optimum form this is conducted by well-trained and experienced practitioners and researchers.

The articles and correspondence columns of our medical journals reveal divergence of opinion as to whether the clinically-minded man and the laboratory-minded man can work in effective unison, or whether, in the alternative, we must await the evolution of a new order of men so gifted and ordained as to be capable of putting on more than a little both ways. Some of us know amongst our research colleagues men for whose abilities and personalities we have the highest regard who would be as much at sea in a hospital ward as we should be amid the tangle and equipment of their laboratories.

Of our liaison colleague, the pathologist, we know much. He is, so to speak, one of us, and is quite often indispensable in matters of clinical diagnosis. We are familiar, too, with his demeanour of benevolent reproach when we attend, shall we say, an occasional *sectio cadaveris* to have our shortcomings pointed out to us. But what about our more recent colleague, the bio-chemist, to whose hands it would seem is entrusted the great objective of revealing the utterly complex factors which constitute, and the conditions which vary resistance to disease and the various infections ? As clinicians we do not know so much of him, but now that he has been summoned from the empyrean to deal with our earth-bound spirits, we confidently hope both to know and to appreciate him, and to introduce him to our patients, if such be his desire. Can our bacteriologists get much farther without some such revelation as that referred to, or will they, without waiting upon the bio-chemist, break through into the unknown themselves and get on with the business ? If so, let them be assured the clinical hosts will follow them hot-foot, and help to at least widen the breach.

Meanwhile, taking the case of septic infection, for example, the clinicians are "milling around" in a space from which there seems, for the time being, to be no escape. And the bacteriologists, to keep us moderately quiet, in the absence of further discoveries, keep on splitting up the streptococci into an ever-increasing number of groups with about as much affinity as exists, for example, between North and South Wales, or between Lancashire and Yorkshire, or even Devon and Cornwall. In our patience we will do our best to possess our souls.

If so much may be submitted regarding the trend of scientific evolution as it affects us, what may be said of the evolution of the legislative frame-work set up by the State during the past twenty years, and in which the profession is required to discharge its duties to the public ?

I suppose that, with one possible exception, there is no other country in the world in which, rightly understood, so much has been done in this direction, and, if I read aright the portents of recent legislation, in which so much more is being and will be done. Presumably it is the relative compactness of the British Isles and the density of their population—two factors which are at once our strength and, in some respects, our weakness—which largely account for this priority of administrative evolution.

The re-alignment of the conditions of general practice brought about by the Health Insurance Acts —a vivid memory to some of us—amounted to something of the nature of a revolution ; bloodless, it is true, but sufficiently disturbing at that. A majority opinion will acclaim those Acts, after eighteen years' experience of their operation, as on the whole successful so far as they go, but equally it will be conceded that they cannot be regarded as other than a veritable torso in relation to a full-bodied National Health Scheme. Fuller developments are, of course, inevitable, and some of them of comparatively minor importance are already available in the form of additional benefits granted by certain of the Approved Societies, but the future has yet to determine the precedence of fundamental additions, such as (*a*) consultant and specialist services, (*b*) institutional treatment, or (*c*) the inclusion of the dependants of insured persons. The policeman's lot may not be a happy one, but it may be beatific as compared with that of the Chancellor of the Exchequer who is called upon to finance any or all of such developments.

But an enactment of the gravest importance to us as members of the medical profession, whether general practitioners or consultants, has arrived from another angle. Whilst our eyes may have been fixed in watchful regard on the movements of the Health Insurance Scheme, the Local Government Act, 1930, camouflaged in the wrappings of a de-rating scheme, has been placed on the statute book by the late Government. Had it been introduced by a Labour Government or — happy dream! — by a Liberal Government, we might have been more concerned; but there it is, and you in Bristol, in common with other centres, have been discussing methods of adjustment to a measure which in its scope and in its effect on all branches of the profession is likely to prove a more profound revolution than even the Insurance Acts.

The sections of the Act which concern us principally are those which impose on the local authorities the duty of making institutional provision for the sick within their boundaries; and under Section 13 in particular, before deciding as to the kind and amount of accommodation required to meet their obligations under the Act, they must consult duly accredited representatives of the voluntary hospitals in their areas. An important consideration is that what has been termed the "taint" of being treated in Poor Law hospitals has been removed, and irrespective of class or category the doors of the hospitals under municipal control are open to all who need the treatment which these hospitals can provide—subject to the recovery of actual *per capita* cost—if necessary by legal process—from those whose financial position can so afford. This proviso at once raises the question of the economic status of the patients in our voluntary hospitals and the consequential difficulties of transfer from a voluntary to a municipal hospital and vice versa. It is obvious, of course, that in any valid scheme of co-operation between the two classes of hospitals facilities for transfer must be provided.

This difficulty is especially exemplified in the case of a patient coming into a voluntary hospital under the unwritten but admitted obligations of one of the group contributions by workmen, which are now so important a factor in the income of the voluntary hospitals. If such a patient be, for any reason, transferred to a municipal hospital (other than an infectious diseases hospital) he incurs the cost of his maintenance therein subject to his proved ability to pay—whereas whilst he was in the voluntary hospital he is exempt from payment of any kind or from any inquiry as to his means. It is clear that difficulty with the group contributors will arise, not to mention that of obtaining the consent of the patient to the transfer. The *per contra* case of transfer from municipal hospital to voluntary hospital of a patient for whom the local authority accepts responsibility will involve payment of consolidated charges, including the cost of maintenance plus a sum which would go to a medical staff fund in accordance with a principle now generally approved. Personally, I do not think that much help can be expected from the establishment of district hospital benefit insurance societies such as have been proposed. To be adequate the premiums would have to be substantial, and neither the local authorities nor the voluntary hospitals would be willing to bear the insurance risk.

For some such reasons as the foregoing, as well as other considerations, it is likely that for some time to come the degree of direct co-operation between the State and the voluntary hospitals will not be great. The grade of treatment in comparable institutions and what is called "the atmosphere" of voluntary hospitals will continue to maintain their popularity, but a very important further attraction to the patients is the low cost—the bulk of them pay little or nothing—and where a large hospital receives a few pence or a few shillings per week from other than ticket or group-contribution patients it does not amount to much. Just how long is this minimal cost to the patients in the typical voluntary hospitals to continue ?

Some will answer, just so long as the legacies, annual subscriptions, special donations and group contributions collectively remain sufficient for the purpose. This is true, but not the whole truth. Incidentally, I think that the group contributions will increase, whilst legacies and individual annual contributions will tend to decrease. But in my judgment a more important determining factor is the unpaid services of the honorary staff. I am very far from even suggesting that the question of the substitution of a salaried service should now be raised. Nothing could be more inopportune. But if and when, owing to the utilization of municipal hospitals, the services of the honorary staff of the voluntary hospitals cease to have their counterpart in private practice a new position will have arisen. Personally I think that epoch is far from imminent, and that those hospitals will be, in any re-arrangement, the more favourably placed which are connected with medical schools.

I am certain that the importance of a large voluntary hospital as a financial mainstay in a commercial centre is not sufficiently realized. Taking the annual value of the worker to the community as so much, the extent to which he and his comrades are treated in such a hospital and returned to work represents to the community a sum equal to the whole income of the hospital manifolded. In other words, the community as a whole invests in the hospital as a going concern a sum equal to the hospital income and receives back yearly in the shape of restored workers the equivalent in money value of a large multiple of that income. An astonishing statement—but capable of actuarial confirmation. Imagine, then, the financial chaos of the city of Bristol were its two great hospitals to close their doors.

In this connection it is readily computed that on a moderate estimate the market value of services rendered by the honorary staff of a general hospital of, say, 350 beds is about £350,000 to £400,000 per annum. I am not submitting that in the alternative that sum divided by the number of the members of the honorary staff would represent their annual incomes. But I do say that herein is the great factor of invisible income upon which, in a given community, the outstanding financial status of the voluntary hospital is based. So long as this remains constant it will be to the economic advantage of employers and workers to continue and, as occasion requires, to increase their contributions. Local authorities, too, will I believe continue, under prevailing conditions, to make those block grants which have in later years become almost general, rather than embark upon schemes involving costly outlay in structure and salaried service. On this latter point, however, much will depend, in different areas, upon the extent to which commitments have already been incurred.

A financial consideration of some importance to our voluntary hospitals is that in other countries, notably in Canada and the United States, much more than in the British Isles, the addition of a wing for private patients has proved to be a sound and even a lucrative investment for the hospital funds, and there is a growing body of opinion that such a development in our midst is overdue. Well-conducted and well-equipped nursing homes and private hospitals would withstand the competition. Others—and there are too many of such—would not.

I am convinced that there is a possibility that the municipal authorities in certain districts may be first in the field.

In my belief, recent legislation notwithstanding, the days of our voluntary hospitals in their priceless services to the community, including medical education and research, are not numbered. Rather is it essential that they shall continue to flourish, and to preserve that sanity of outlook and enterprise which, as some of us know from personal observation in other lands, has endeared and ennobled them in the view of our colleagues the world over.

Some of the points I have dealt with—though all too scantily—Mr. President, are unquestionably of deep interest to all of us. So much so that in order to give myself the advantage of knowing the views of members present I am tempted to beg you to depart from precedent and to allow some general discussion to follow.

Once again, sir, may I express my warm thanks for the honour of being invited to address the Society.

